# Relationship between platelet activation markers and spontaneous abortion: A meta-analysis

**DOI:** 10.1515/biol-2022-0485

**Published:** 2022-12-15

**Authors:** Hua Gao, Hui-Juan Ma, Ying-Jia Li, Yun Li, Ji-Rong Zhang

**Affiliations:** Department of Outpatient, Lanzhou University Second Hospital, Lanzhou University Second Clinical Medical College, Lanzhou University, Lanzhou, 730030, China; Department of Anesthesiology and Surgery, Lanzhou University Second Hospital, Lanzhou University Second Clinical Medical College, Lanzhou 730030, China

**Keywords:** MPV, PDW, pregnancy loss, miscarriage

## Abstract

Numerous studies have indicated that mean platelet volume (MPV) and platelet distribution width (PDW) were correlated with spontaneous abortion (SAB), but the results were contradictory. Hence, we performed a meta-analysis to assess the association of MPV and PDW with SAB. We systematically searched on China National Knowledge Internet, PubMed, and Embase up to May 2022 to retrieve eligible studies. The synthesized standard mean difference (SMD) with a 95% confidence interval (CI) was used to evaluate the correlation of MPV and PDW with SAB. A total of 20 studies were finally included in this meta-analysis. The pooled analysis results showed that PDW was markedly increased in patients with SAB versus women with a healthy pregnancy (SMD = 1.03; 95% CI: 0.51–1.54; *p* = 0.0001), while there were no significant differences in MPV between women with SAB and those with healthy pregnancy (SMD = 0.19; 95% CI: −0.26 to 0.65; *p* = 0.40). Therefore, PDW may serve as a potential marker for predicting SAB. However, homogeneous and multiethnic studies with larger sample sizes are warranted to validate our findings due to several limitations in this meta-analysis.

## Graphical abstract

**Figure:**
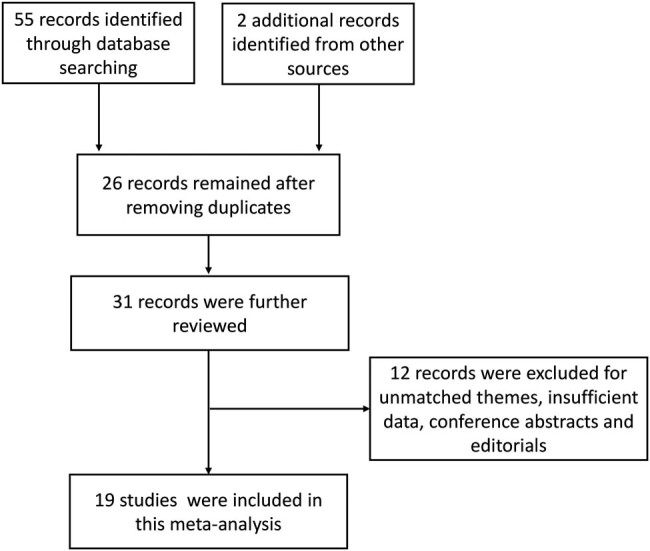

## 1 Introduction

Spontaneous abortion (SAB) is known to be the most frequent pregnancy complication, affecting approximately 15% of all pregnancies [1]. In particular, approximately 80% of all miscarriages occur in the first trimester. Solid evidence in the literature indicates that chromosomal anomalies, endocrine disorders, immunological causes, anatomic abnormalities, uterine anomalies, and infectious events are important causes of SAB [2,3,4,5]. However, the pathophysiology of spontaneous pregnancy loss is rather intricate and has never been fully understood [6]. Moreover, there is a lack of effective methods for predicting spontaneous miscarriage in clinical practice [7,8].

The compatible contact between the placenta and maternal circulation is a vital determinant for a successful pregnancy [9]. Conversely, thrombophilia, under which the tendency for venous thrombosis largely increases, has been considered to correlate with pregnancy loss [9,10]. Once microemboli form in the uteroplacental circulation in pregnant women with thrombophilia, a placental microenvironment conducive to fetal development is disturbed, which causes abnormal nutritional supply and inflammation, and consequently results in miscarriage [9,10,11]. Platelet activation is a pivotal event during thrombosis, which leads to the release of a mass of β-thrombo-globulin and soluble platelet P-selectin, largely increasing the risk of thrombophilia [12]. For this reason, β-thrombo-globulin and soluble platelet P-selectin have been proposed to serve as potential predictors of spontaneous miscarriage. However, the measurements of the two makers are time-consuming and expensive, thereby limiting their application in clinical practice [13]. Thus, it is imperative to identify easily accessible and cost-effective laboratory markers for predicting spontaneous pregnancy loss.

Mean platelet volume (MPV) and platelet distribution width (PDW), which are routinely detected in clinical laboratories, have been determined as parameters of platelet activation [14,15]. Intriguingly, numerous studies have investigated the association of MPV and PDW with spontaneous pregnancy loss, but the results were conflicting [7,8,13,16,17,18,19]. Therefore, herein we further evaluated the relationship of MPV and PDW to SAB through a systematic review and meta-analysis of published literature, to assess the potential of MPV and PDW as markers of predicting SAB.

## 2 Materials and methods

### 2.1 Ethical approval and informed consent

The conducted research is not related to either human or animal use, so ethical approval and informed consent are unnecessary.

### 2.2 Search strategy

Two researchers independently conducted the literature collection by searching PubMed, Embase, and China National Knowledge Internet up to May 2022. Search terms included “MPV, PDW or ” and “abortion or miscarriage or pregnancy loss.” In addition, the references of retrieved studies were screened to identify additional eligible studies omitted by electronic search. The detailed search strategies for each database were presented in Supplement 1.

### 2.3 Eligibility criteria

The studies synchronously satisfying the inclusion criteria mentioned below were eligible: (1) had a full-text organization structure; (2) investigated the association of MPV or PDW with SAB and reported the mean MPV or PDW and their corresponding SD in patients and women with healthy pregnancy; and (3) published in English or Chinese. For studies with overlapping data, the study with the larger sample size was selected. In contrast, studies were excluded if they met any of the following criteria: (1) they did not assess the association of MPV or PDW with SAB; (2) they did not provide data about the mean MPV or PDW and their corresponding SD; or (3) they were academic dissertations, review articles, editorial articles, meta-analyses, or meeting abstracts.

### 2.4 Quality assessment

The Newcastle-Ottawa scale (NOS) was applied to appraise study quality [20]. The NOS covers eight items for observational studies and is categorized into three dimensions: selection, comparability and exposure. The “star” rating system is utilized to grade the methodological quality. A maximum of four stars are used to rate the selection dimension, two stars are used to rate the comparability dimension, and three stars are used to rate the exposure dimension. The total score ranges from 0 (worst) to 9 stars (best), and the quality of each study is assessed as low (0–3), moderate (4–6), or high (7–9).

### 2.5 Data extraction

Two researchers extracted the data independently based on the inclusion criteria. Extracted data were fed into a collection table and examined by a third author. Disagreements were removed via discussion. In this meta-analysis, the extracted data included the study author, publication year, country, sample size, age of pregnant women, gestational age, type of abortion, mean MPV value, mean PDW value, and SD of MPV and PDW.

### 2.6 Statistical analysis

The statistical analysis was conducted using STATA version 12.0 (Stata Corporation, College Station, TX, USA) and RevMan version 5.3 software. The standard mean difference (SMD) and its 95% confidence interval (CI) were utilized to evaluate the pooled MPV and PDW values. When the SMD was above 0 and the 95% CI did not cross 0, the MPV or PDW value was considered to be increased in the SAB group versus the control group. Otherwise, the combination of SMD <0 and the 95% CI not crossing 0 indicated a decrease in the MPV or PDW value in the SAB group versus the control group. Additionally, when the 95% CI crossed 0, the MPV or PDW value was considered to be equal between the two groups. The Cochrane *Q* test (*χ* ^2^) and *I* ^2^ test were used to assess the statistical heterogeneity among the studies. The random-effect model was selected to calculate the SMD when there was significant heterogeneity. *p* < 0.05 was considered to indicate a statistically significant difference. Subgroup analyses and meta-regression analyses based on sample size, country, age of pregnant women, gestational age, and recurrent miscarriage were carried out to explore the sources of heterogeneity [21]. Sensitivity analyses were performed by excluding one study in each step. The Begg funnel plot and Egger’s test were applied to assess publication bias [20].

## 3 Results

### 3.1 Study characteristics

**Figure 1:**
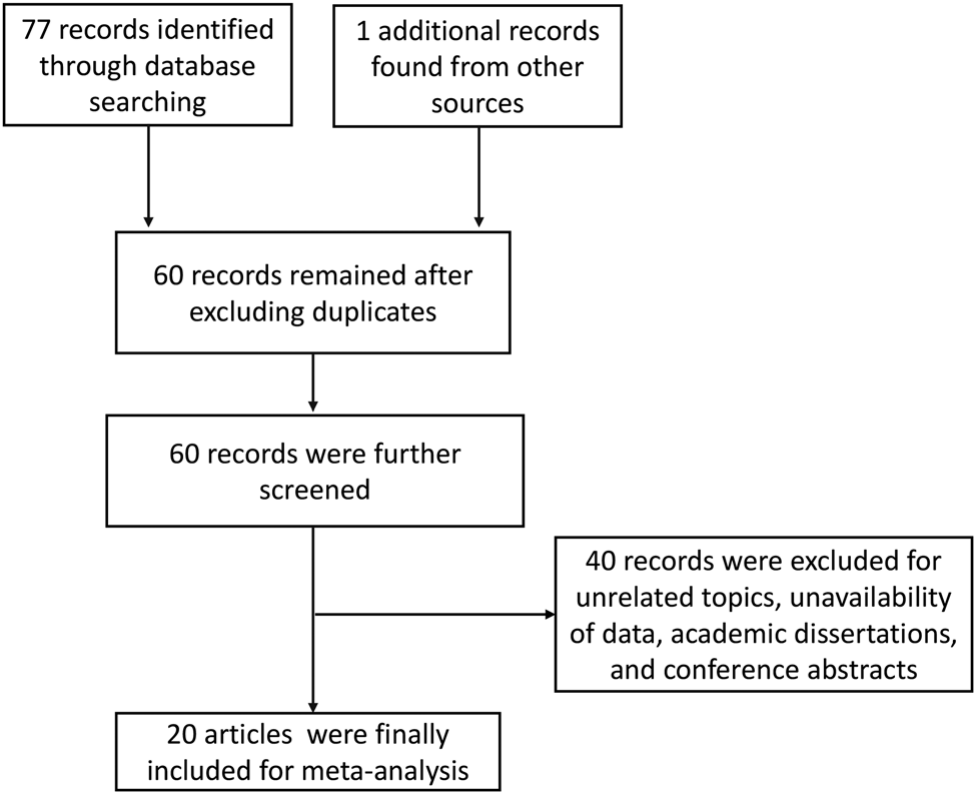
Flowchart of study search and identification.

Through electronic and manual searches, we first obtained 77 articles. Then, academic dissertations, duplicate articles, studies with unmatched themes, studies lacking data about MPV or PDW, and articles without full text were excluded; thus, 57 studies were excluded, and 20 studies were finally included in this meta-analysis [7,8,13,16,17,18,19,22,23,24,25,26,27,28,29,30,31,32,33,34]. The flowchart of the study search and selection process is presented in Figure 1. The publication years of the included studies ranged from 2013 to 2022. Most of the studies (*n* = 14) came from Turkey, and the others were conducted in China. The sample size of the included studies ranged from 80 to 2192. A total of 13 studies focused on recurrent miscarriage. Among the included studies, 18 studies reported MPV measurements, while 14 studies reported PDW. The methodological quality of the studies was evaluated by NOS, and the scores ranged from 6 to 7, implying that the included studies had a relatively acceptable quality. The detailed information about study characteristics is summarized in Table 1.

**Table 1:** The main characteristics of included studies

Author/year	Country	Case number		Age (year)		Recurrent abortion	Gestational age	Mean platelet volume (fl)				Platelet distribution width (%)				Quality score
		Spontaneous abortion	Heathy pregnancy	Spontaneous abortion	Heathy pregnancy			Spontaneous abortion		Heathy pregnancy		Spontaneous abortion		Heathy pregnancy		
								Mean	SD	Mean	SD	Mean	SD	Mean	SD	
Akdemir et al., 2013	Turkey	51	64	32.1 ± 3.5	29.4 ± 4.0	Yes	<13 week	8.1	1.1	8.2	1.5	17.8	1	18	1.2	7
Akin et al., 2016	Turkey	78	91	30.3 ± 6.8	28.1 ± 5.4	NR	<13 week	9.5	1.1	8.8	1	16.3	2.6	16.5	1.6	7
Ata et al., 2020	Turkey	100	100	28.1 ± 4.0	27.1 ± 5.2	NR	<13 week	10.01	1.25	10.6	0.85	NR	NR	NR	NR	6
Aynıoglu et al., 2016	Turkey	208	95	31 ± 3.8	34 ± 5.5	Yes	<20 week	8.1	2.37	8.9	0.97	NR	NR	NR	NR	7
Bas et al., 2018	Turkey	173	245	31.88 ± 6.43	30.15 ± 5.62	NR	<13 week	6.72	2.41	8.67	0.94	NR	NR	NR	NR	6
Biyik et al., 2020	Turkey	40	40	29.27 ± 6.84	28.37 ± 5.13	NR	<13 week	9.07	1.48	9.72	1.33	16.65	0.79	16.3	0.73	6
Dundar et al., 2015	Turkey	60	60	27.0 ± 5.2	27.6 ± 5.3	Yes	NR	10.9	1.1	10.5	0.9	17.5	3.8	15.9	0.7	7
Erdem et al., 2020	Turkey	50	60	29.8 ± 5.8	28.7 ± 5.2	Yes	<20 week	9.8	1.7	8.3	1.8	15.7	1.8	14.9	2.5	6
Kaplanoglu et al., 2015	Turkey	305	168	27.16 ± 4.59	27.62 ± 4.41	NR	<20 week	8.99	1.47	9.66	1.64	17.31	2.99	17.78	3.28	6
Li and Ma, 2017	China	28	40	30 ± 2.75	28 ± 3.75	Yes	<20 week	10.86	2.07	9.52	1.83	15.65	2.32	13.56	2.04	6
Li et al., 2020	China	80	80	32 ± 5	30 ± 4	Yes	<28 week	11.04	1.48	10.49	1.28	16.55	0.46	16.36	0.4	6
Lu et al., 2022	China	215	160	26.7 ± 3.8	26.5 ± 3.9	Yes	NR	12.12	1.32	10.35	1.31	17.57	0.45	16.34	0.37	7
Oğlak and Aydın, 2020	Turkey	137	148	23 ± 2.83	26 ± 2.67	NR	<13 week	8.8	0.68	8.6	0.67	15.8	0.37	15.2	0.77	7
Qian et al., 2020	China	202	90	29.2 ± 4.5	28.8 ± 5.1	Yes	<28 week	NR	NR	NR	NR	16.39	3.9	11.28	2.03	7
Uysal et al., 2015	Turkey	50	50	26.18 ± 3.63	26.57 ± 3.74	Yes	NR	8.06	1.56	7.72	1.1	17.85	1.75	17.96	2.27	7
Mete Ural, 2014	Turkey	74	208	31 ± 2.7	32 ± 1.9	Yes	<13 week	7.99	1.02	7.98	1.24	19.89	1.21	18.59	1.77	7
Yakıştıran et al., 2021	Turkey	193	164	30.6 ± 6.8	27.8 ± 5.6	NR	<13 week	8.4	6.5	8.3	1.0	NR	NR	NR	NR	6
Yilmaz et al., 2013	Turkey	120	120	29.07 ± 2.81	28.53 ± 3.5	Yes	NR	9.45	1.09	7.63	0.52	NR	NR	NR	NR	6
Yu et al., 2020	China	206	92	32.36 ± 4.55	32.87 ± 3.82	Yes	<28 week	8.53	0.92	8.8	0.61	NR	NR	NR	NR	7
Zhang et al., 2018	China	780	1412	31 ± 5	31 ± 7	Yes	<28 week	NR	NR	NR	NR	15.5	2.5	12.8	2.5	6

NR: not reported; SD: standard deviation.

### 3.2 Meta-analysis of the association between PDW and SAB

**Figure 2:**
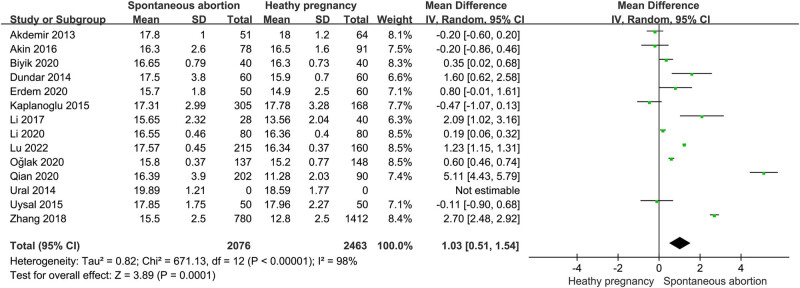
Meta-analysis of the association between PDW and spontaneous abortion.

Fourteen studies involving 2,076 patients and 2,303 subjects with healthy pregnancy explored the relationship of PDW with spontaneous miscarriage [13,16,17,18,19,22,25,26,27,28,29,30,31,32]. Mean PDW values and SD between patients with miscarriage and healthy counterparts were subjected to meta-analysis. In view of the significant heterogeneity (*I* ^2^ = 98.0; *p* < 0.00001), we used random-effect models to perform the meta-analysis for PDW. The result of the overall pooled analysis showed that the PDW level was markedly increased in the SAB group versus the healthy pregnancy group (SMD = 1.03; 95% CI: 0.51–1.54 [Figure 2]). Then, subgroup analyses and meta-regression analyses by sample size, country, age of pregnant women, gestational age, and recurrent miscarriage were conducted to the sources of heterogeneity (Table 2). We found a positive correlation between PDW level and SAB in the subgroups of sample size (*N* > 200 and *N* ≤ 200), China, age of pregnant women (≤30 years), gestational age (<28 weeks), and recurrent abortion, but not in the other subgroups. Meanwhile, significant heterogeneity was still observed in all the subgroup analyses. However, the results of meta-regression analyses suggested that the study country may be one of the major sources of heterogeneity for the pooled estimation of the correlation between PDW and SAB.

**Table 2:** Subgroup and meta-regression analyses for the correlation of PDW with spontaneous abortion

Stratified study	No. of studies	Pooled SMD (95% CI)	*p*-value	Heterogeneity		Regression		
				*I* ^2^ (%)	*p*-Value	*I* ^2^ (%)	Adj *R* ^2^	*p*-value
1 Sample size						96.97	8.99	0.16
*N* ≤ 165	7	0.352 (0.086–0.619)	0.01	69.6	<0.01			
*N* > 165	7	1.003 (0.375–1.631)	<0.01	98.4	<0.01			
2 Country						95.23	38.75	0.012
China	5	1.388 (0.661–2.116)	<0.01	97.6	<0.01			
Turkey	9	0.302 (−0.027 to 0.631)	0.072	90.4	<0.01			
3 Age						97.35	−1.29	0.374
>30 years	5	0.42 (−0.112 to 0.953)	0.122	95.9	<0.01			
≤30 years	9	0.845 (0.155 to 1.535)	0.016	97.7	<0.01			
4 Gestational age						96.23	13.17	0.119
<13 weeks	4	0.296 (−0.313 to 0.906)	0.34	92.7	<0.01			
<20 weeks	4	0.472 (−0.092 to 1.036)	0.101	92.6	<0.01			
<28 weeks	3	1.012 (0.562 to 1.461)	<0.01	91.8	<0.01			
NA	3	1.162 (−0.731 to 3.055)	0.229	98.9	<0.01			
5 Recurrent abortion						96.43	2.52	0.279
Yes	10	0.85 (0.37–1.329)	<0.01	96.8	<0.01			
NR	4	0.295 (−0.301 to –0.891)	0.332	94.7	<0.01			

NR: not report.

### 3.3 Meta-analysis of the association between MPV and SAB

**Figure 3:**
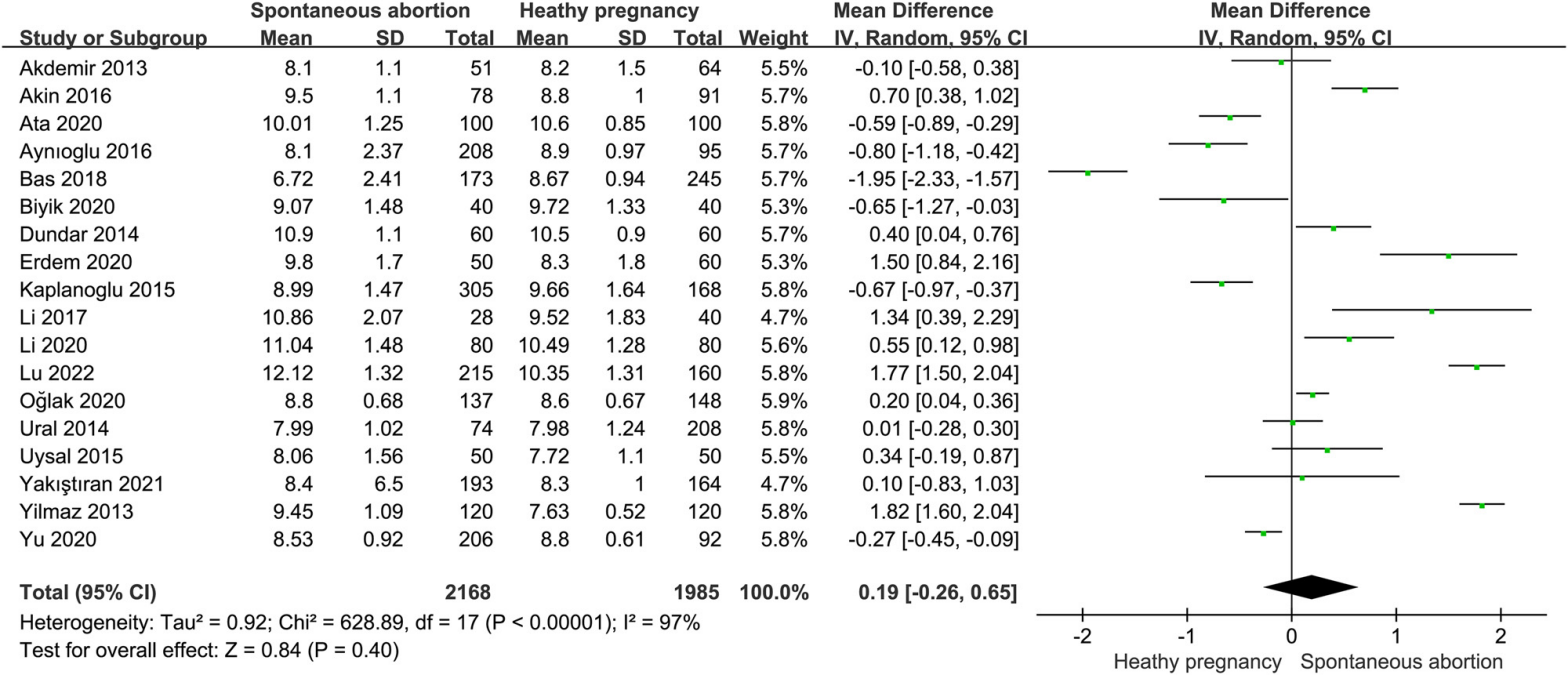
Meta-analysis of the association between MPV and spontaneous abortion.

A total of 18 studies involving 1,953 women with SAB and 1,825 subjects with healthy pregnancy investigated the association between MPV and SAB [7,8,13,16,17,18,19,22,23,24,25,27,28,29,30,31,33,34]. The mean values of MPV and SD between patients with spontaneous miscarriage and women with healthy pregnancy were subjected to meta-analysis. Considering the significant heterogeneity, the random-effect models (*I* ^2^ = 97.0%; *p* < 0.00001) were applied in the meta-analysis for MPV. The pooled result showed that there were no significant differences in MPV levels between patients with SAB and control subjects (SMD = 0.19; 95% CI: −0.26 to 0.65 [Figure 3]). To minimize the effect of confounding factors on the pooled result and to investigate the potential sources of heterogeneity, we conducted subgroup analyses and meta-regression analyses based on sample size, country, age of pregnant women, gestational age, and recurrent miscarriage (Table 3). In general, no significant differences in MPV between women with SAB and subjects with healthy pregnancies were found in all the subgroup analyses, except the subgroup of gestational age (NR). Additionally, significant heterogeneity still existed in all the subgroup analyses, indicating that those confounding factors above may not the major sources of the heterogeneity. Nevertheless, the results of meta-regression analyses supported that the gestational age of SAB may partly explain the heterogeneity for the pooled estimation of the correlation between MPV and SAB.

**Table 3:** Subgroup and meta-regression analyses for the correlation of MPV with spontaneous abortion

Stratified study	No. of studies	Pooled SMD (95% CI)	*p*-Value	Heterogeneity		Regression		
				*I* ^2^ (%)	*p*-Value	*I* ^2^ (%)	Adj *R* ^2^	*p*-value
1 Sample size						96.98	−6.14	0.839
*N*＞200	9	0.164 (−0.427 to 0.755)	0.586	98.3	<0.01			
*N* ≤ 200	9	0.238 (−0.104 to 0.579)	0.173	97.8	<0.01			
2 Country						96.66	−0.48	0.354
China	4	0.527 (−0.31 to 1.364)	0.217	96.9	<0.01			
Turkey	14	0.108 (−0.293 to 0.509)	0.598	96.6	<0.01			
3 Age						96.47	8.94	0.128
>30 years	8	−0.111 (−0.51 to 0.288)	0.586	94.7	<0.01			
≤30 years	10	0.452 (−0.114 to 1.018)	0.117	97.2	<0.01			
4 Gestational age						95.23	30.17	0.013
<13 weeks	7	−0.178 (−0.668 to 0.312)	0.476	95.6	<0.01			
<20 weeks	5	0.111 (−0.342 to 0.564)	0.631	92	<0.01			
<28 weeks	2	0.031 (−0.675 to 0.736)	0.932	92	<0.01			
NR	4	1.039 (0.246–1.833)	0.01	96	<0.01			
5 Recurrent abortion						96.14	16.87	0.054
Yes	11	0.479 (−0.009 to 0.967)	0.054	96.4	<0.01			
NR	7	−0.231 (−0.68 to 0.219)	0.315	95.7	<0.01			

NR: not report.

### 3.4 Sensitivity analyses and publication bias

**Figure 4:**
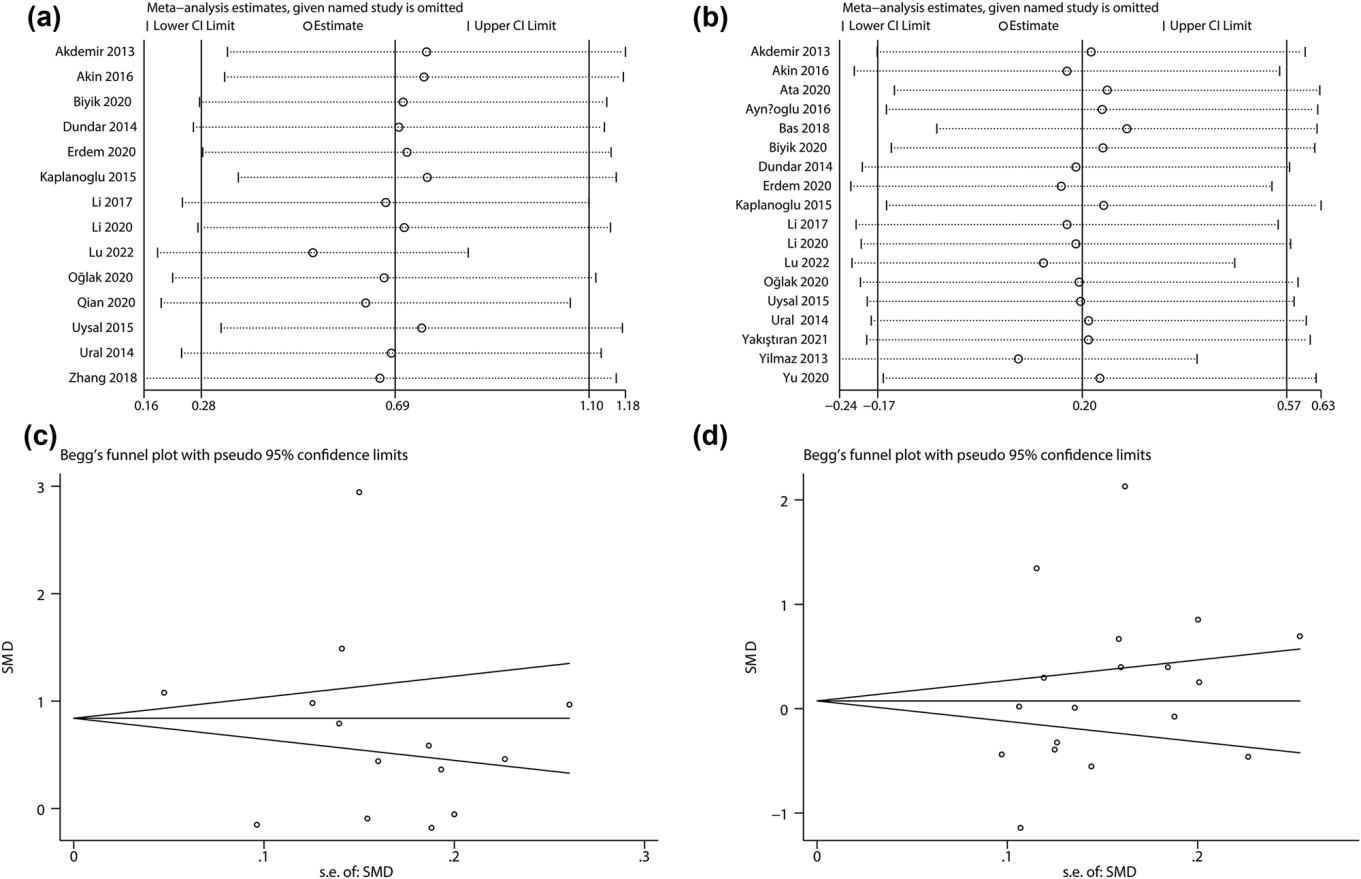
Sensitivity analysis for (a) PDW and (b) MPV; funnel plot describing publication bias of (c) PDW and (d) MPV.

To assess the robustness of meta-analysis, sensitivity analysis was performed by omitting eligible studies one by one and then pooling the data of the remaining studies. The results showed that the overall effect size of PDW and MPV exhibited no significant alterations with the exclusion of any study (Figure 4a and b). Additionally, we applied Begg’s funnel plot and Egger’s test to evaluate the publication bias. As shown in Figure 4c and d, Begg’s funnel plots for PDW and MPV were symmetric, indicating the absence of substantial publication bias. Furthermore, the *p* values of Egger’s test for PDW and MPV were 0.413 and 0.181, respectively, which further confirmed that no significant publication bias existed in this meta-analysis.

## 4 Discussion

Pregnancy loss represents a serious reproductive health problem, which brings huge emotional and physical damage to thousands of families [35]. The etiology of spontaneous pregnancy loss is multifactorial and remains largely unknown, and there is a lack of an effective method of predicting miscarriage in clinical practice [35]. A growing number of studies have found that PDW and MPV, two simple, easily measurable, and cost-effective parameters, were markedly increased in women with SAB versus in counterparts with healthy pregnancies, suggesting that they may serve as tools to predict spontaneous miscarriage [13,17,22]. However, some conflicting results on this topic were also reported [7,8,16,25,31]. Therefore, the association of PDW and MPV with spontaneous pregnancy loss should be further assessed. To our best knowledge, this study is the first systematic review and meta-analysis to comprehensively evaluate the relationship of PDW and MPV to spontaneous miscarriage. We found that PDW value was much higher in patients with SAB than that in women with normal pregnancy (SMD = 1.03; 95% CI: 0.51–1.54; *p* = 0.0001), whereas there were no significant differences in MPV value (SMD = 0.19; 95% CI: −0.26 to 0.65; *p* = 0.40).

The concentrations of coagulation factors are often altered during pregnancy, leading to a state of hypercoagulation [36]. Moreover, platelet activity is also increased in pregnant women, and activated platelets are capable of producing a rich number of prothrombotic meditators, such as thromboxane A2, serotonin, P-selectin, and glycoprotein, thereby contributing to hypercoagulatory state [37]. This prothrombotic state will bring about uteroplacental vascular thrombosis, which is a major cause of pregnancy loss [38]. PDW, a parameter for describing the variation in platelet size, has been determined as a specific marker of platelet activation [25]. For these reasons, many studies have been performed to explore the association of PDW with spontaneous miscarriage. Some studies showed that the PDW value was much higher in women with SAB than in subjects with healthy pregnancies, indicating the potential of PDW as a marker of predicting pregnancy loss [13,26,28,31,32]. However, other studies reported no significant relationship between PDW and spontaneous miscarriage [19,22,25,27]. The conflicting results may be partly caused by the limited sample size of those individual studies. To remove this influence, we carried out a meta-analysis of the literature studies on this topic. Consistent with some previous research articles [13,26,28,31,32], our overall pooled analysis found a higher PDW value in women with spontaneous miscarriage compared to those with healthy pregnancies. There was significant heterogeneity across the included studies referring to PDW, which may challenge the validity of our meta-analysis. Hence, to account for the heterogeneity, we performed the subgroup analyses based on sample size, country, biological age, gestational age, and miscarriage type. However, substantial heterogeneity still existed in each subgroup, suggesting that these factors might not be the major sources of heterogeneity. Additionally, PDW values were significantly higher in patients with spontaneous miscarriage versus in women with healthy pregnancy in subgroups of sample size (*N* > 200 and *N* ≤ 200), China, biological age (≤30 year), gestational age (<28 week), but not in the other subgroups. The limitation of case numbers in individual subgroups may be partly responsible for these contradictory results. Of note, our sensitivity analysis showed that the overall estimation of PDW did not substantially alter with a single study sequentially excluded, and no evident publication bias for studies referring to PDW was identified, which suggested the validity of our meta-analysis to a degree. In addition to the hypercoagulation state, an excessive maternal inflammatory response is another crucial cause of spontaneous pregnancy loss [17]. The platelets in activated form can also promote inflammatory response through releasing small molecular meditators that activate other immune cells, notwithstanding their major functions are hemostasis and coagulation [39]. Remarkably, PDW has been identified as a systemic inflammatory marker, and its value is positively correlated with the activity of many inflammatory diseases [25,39]. Hence, the correlation between PDW and systematic inflammation may also explain the finding of our meta-analysis at least partly.

MPV is a parameter of evaluating platelet size and has also been identified as a marker of platelet activation [40] and systematic inflammation [39]. A large number of studies have demonstrated that incremental MPV level was associated with a higher risk of venous thromboembolism in the context of multiple diseases [41,42]. Meanwhile, MPV level was found to be elevated in various inflammatory disorders and positively correlate with the activity and severity of diseases [43,44,45,46]. Furthermore, many research studies have also been conducted to investigate the relationship between MPV and spontaneous miscarriage. However, the results on this topic were conflicting. For example, Akin et al. [22] suggested that a higher MPV value was correlated with spontaneous pregnancy loss, whereas Uysal et al. [19] found no significant association between MPV and SAB, and Bas et al. [24] and Ata et al. [7] even reported that there was an inverse link between MPV value and pregnancy loss. In this study, our overall pooled analysis demonstrated that there were no significant differences in MPV between women with spontaneous miscarriage and subjects with healthy pregnancies. To minimize the influence of confounding factors on the pooled estimation and interpret the heterogeneity, we further conducted subgroup analyses by sample size, country, biological age, gestational age, and miscarriage type. In accordance with the overall pooled estimation, all the subgroup analyses showed that MPV values were similar in patients with SAB and women with healthy pregnancies. In addition, sensitivity analysis by omitting a single study in each step showed that the combined analysis of the correlation between MPV and spontaneous miscarriage was stable. Moreover, Begg’s and Egger’s tests detected no significant publication bias across included studies reporting MPV. Overall, these results supported that our meta-analysis of the association between MPV and spontaneous pregnancy loss was reliable and robust. Superficially, the results contradicted the potential of MPV as a marker of platelet activation and systematic inflammation. In fact, MPV values may be influenced by multiple factors in women with pregnancy loss. MPV can be directly affected by platelet distension [47], under which condition an increment in MPV value may be detected, though platelet activation does not exist. Meanwhile, it has been revealed that activated platelets with larger volumes can migrate to the region in early gestational periods [7,13], consequently leading to a decreased MPV level in the circulation of women with pregnancy loss. When these factors that can influence MPV value coexist in pregnant women, the measured value of MPV may depend on which factor plays the major role and fail to truly reflect the platelet activity. Therefore, MPV alone may not be reliable for predicting pregnancy loss, although it correlates with platelet activation. Of note, it has been proposed that the combination of PDW and MPV could reflect the activation of coagulation more accurately. In the future, it will be of interest to explore the potential of the combination of PDW and MPV in predicting spontaneous pregnancy loss.

There are several limitations in our meta-analysis, so the results should be interpreted cautiously. First of all, the clinical heterogeneity among the included studies exists in this meta-analysis, such as definitions of spontaneous miscarriage, age of patients and control subjects, gestational age, thromboembolism-precipitating risk, methods of measuring PDW and MPV, and testing time. Thus, it is hard to exclude the possibility that the pooled results could be biased by heterogenic factors. Second, all of the included studies collected data with the retrospective method, which may be accompanied by a high risk of selection bias. Third, this study only compared the levels of PDW between women with pregnancy loss and healthy subjects, while the cut-off values of high PDW levels were not determined due to the unavailability of relevant data in the included studies. This drawback makes it difficult to use PDW as a clinical tool to predict spontaneous pregnancy loss. Fourth, publication bias might exist since only studies published in English and Chinese were considered in this meta-analysis, notwithstanding the funnel plot and Egger’s test indicating no evident publication bias. Fifth, all the included studies only enrolled Chinese or Turks, so it is arbitrary to generalize our conclusion to other ethnicities. Last but not least, trial sequential analysis (TSA) can help to evaluate whether the least sample size required for obtaining a stable and robust conclusion is enough [48]. Of note, TSA is just suitable for the meta-analysis of studies that assesses the causal correlation of the risk factors with outcomes using relative risk (RR) and odds risk (OR) with 95% CIs. However, in this meta-analysis, we only compared the values of PDW and MPV between patients with SAB and subjects with a healthy pregnancy, and the OR and RR for evaluating the correlation of PDW and MPV with spontaneous miscarriage between PDW were not reported in the included studies. Therefore, TSA could not be performed in the present study.

## 5 Conclusion

In summary, PDW was markedly increased in patients with SAB versus women with healthy pregnancies, while there were no significant differences in MPV between patients with SAB and women with healthy pregnancies. Therefore, PDW may be used as a potential marker for predicting SAB. However, homogeneous and multiethnic studies with larger sample sizes are warranted to validate our findings due to several limitations in this meta-analysis.

## Supplementary Material

Supplementary Material

## References

[1] Ghazanfarpour M, Kashani ZA, Pakzad R, Abdi F, Rahnemaei FA, Akbari PA, et al. Effect of electromagnetic field on abortion: a systematic review and meta-analysis. Open Med (Wars). 2021;16(1):1628–41. 10.1515/med-2021-0384 PMC8569282 34761114

[2] Yifu P, Lei Y, Shaoming L, Yujin G, Xingwang Z. Sperm DNA fragmentation index with unexplained recurrent spontaneous abortion: A systematic review and meta-analysis. J Gynecol Obstet Hum Reprod. 2020;49(10):101740. 10.1016/j.jogoh.2020.101740 32348878

[3] Zhu W, Zheng H, Liu J, Cai J, Wang G, Li Y, et al. The correlation between chronic exposure to particulate matter and spontaneous abortion: a meta-analysis. Chemosphere. 2022;286(Pt 2):131802. 10.1016/j.chemosphere.2021.131802 34426134

[4] Fu YY, Ren CE, Qiao PY, Meng YH. Uterine natural killer cells and recurrent spontaneous abortion. Am J Reprod Immunol. 2021;86(2):e13433. 10.1111/aji.13433 33896061

[5] Li D, Zheng L, Zhao D, Xu Y, Wang Y. The role of immune cells in recurrent spontaneous abortion. Reprod Sci. 2021;28(12):3303–15. 10.1007/s43032-021-00599-y PMC8186021 34101149

[6] Lou C, Goodier JL, Qiang R. A potential new mechanism for pregnancy loss: considering the role of LINE-1 retrotransposons in early spontaneous miscarriage. Reprod Biol Endocrinol. 2020;18(1):6. 10.1186/s12958-020-0564-x PMC6971995 31964400

[7] Ata N, Kulhan M, Kulhan NG, Turkler C. Can neutrophil-lymphocyte and platelet-lymphocyte ratios predict threatened abortion and early pregnancy loss? Ginekol Pol. 2020;91(4):210–5. 10.5603/GP.2020.0042 32374021

[8] Yakıştıran B, Tanacan A, Altınboğa O, Yücel A. Can derived neutrophil-to-lymphocyte ratio, platelet-to-lymphocyte ratio, and delta neutrophil index predict spontaneous abortion? Z Geburtshilfe Neonatol. 2021;225(5):418–22. 10.1055/a-1363-2855 33530116

[9] Kaymaz E, Gun BD, Genc GC, Kokturk F, Ozmen KG. May the morphological findings in the first-trimester abortion materials be indicative of inherited thrombophilia? J Obstet Gynaecol Res. 2020;46(11):2261–71. 10.1111/jog.14419 32840015

[10] Bagot CN, Pavord S, Hunt BJ. Managing venous thromboembolic risk in women undergoing spontaneous or induced early pregnancy loss: a consensus statement from the British Society of Haematology Obstetric Haematology Special Interest Group. Br J Haematol. 2020;190(1):115–8. 10.1111/bjh.16496 32026460

[11] Bianco M, Mantovani S, D’Agostino FG, Bassi M, Amore D, Cagnetti S, et al. Deep venous thrombosis and abortion: an unusual clinical manifestation of severe form of pectus excavatum. Gen Thorac Cardiovasc Surg. 2021;69(5):897–901. 10.1007/s11748-020-01583-0 PMC8058001 33502689

[12] Kaur S, Singh A, Kaur J, Verma N, Pandey AK, Das S, et al. Upregulation of cytokine signalling in platelets increases risk of thrombophilia in severe COVID-19 patients. Blood Cells Mol Dis. 2022;94:102653. 10.1016/j.bcmd.2022.102653 PMC8832951 35180460

[13] Dundar O, Pektas MK, Bodur S, Bakir LV, Cetin A. Recurrent pregnancy loss is associated with increased red cell distribution width and platelet distribution width. J Obstet Gynaecol Res. 2015;41(4):551–8. 10.1111/jog.12600 25370870

[14] Cai N, Chen ZQ, Tao M, Fan WT, Liao W. Mean platelet volume and red blood cell distribution width is associated with prognosis in premature neonates with sepsis. Open Med (Wars). 2021;16(1):1175–81. 10.1515/med-2021-0323 PMC8389506 34514164

[15] Awad A, Elnemr S, Hodeib H, El Amrousy D. Platelet activation markers in children with pulmonary arterial hypertension associated with congenital heart disease. Pediatr Cardiol. 2022;43(6):1264–70. 10.1007/s00246-022-02847-7 PMC9293825 35234994

[16] Akdemira N, Cevrioglua AS, Ozdena S, Kurua B, Bilira F, Bilirb C. Platelet indices and blood groups in early recurrent miscarriage: a study in pregnant women. J Clin Gynecol Obstet. 2013;2(1):27–30.

[17] Erdem ZS, Cayir Y, Kosan Z, Erdem HB. Is there any relation between recurrent miscarriage and complete blood count values? A case-control study. Konuralp Tip Dergisi. 2020;12(1):39–43.

[18] Mete Ural U, Bayoğlu Tekin Y, Balik G, Kir Şahin F, Colak S. Could platelet distribution width be a predictive marker for unexplained recurrent miscarriage? Arch Gynecol Obstet. 2014;290(2):233–6. 10.1007/s00404-014-3192-x 24619190

[19] Uysal A, İncebıyık A, Hacıvelioğlu S, Gencer M, Güngör A, Coşar E. Is there any relationship between platelet functions, red cell distribution width and recurrent pregnancy loss? J Clin Anal Med. 2015;6(2):149–51.

[20] Zhang YL, Chen P, Guo Y, Zhang YJ. Clinical value of SIRT1 as a prognostic biomarker in esophageal squamous cell carcinoma, a systematic meta-analysis. Open Med (Wars). 2022;17(1):527–35. 10.1515/med-2022-0454 PMC8924434 35350833

[21] Rahnemaei FA, Pakzad R, Amirian A, Pakzad I, Abdi F. Effect of gestational diabetes mellitus on lipid profile: A systematic review and meta-analysis. Open Med (Wars). 2022;17(1):70–86. 10.1515/med-2021-0408 PMC8678474 34993347

[22] Akin MN, Kasap B, Yuvaci HU, Turhan N. Association between platelet indices and first trimester miscarriage. Blood Coagul Fibrinolysis. 2016;27(5):526–30. 10.1097/MBC.0000000000000445 26569515

[23] Aynıoglu O, Isık H, Sahbaz A, Harma MI, Isık M, Kokturk F. Can plateletcrit be a marker for recurrent pregnancy loss? Clin Appl Thromb Hemost. 2016;22(5):447–52. 10.1177/1076029614565882 25550079

[24] Bas FY, Tola EN, Sak S, Cankaya BA. The role of complete blood inflammation markers in the prediction of spontaneous abortion. Pak J Med Sci. 2018;34(6):1381–5. 10.12669/pjms.346.15939 PMC6290208 30559789

[25] Biyik I, Albayrak M, Keskin F. Platelet to lymphocyte ratio and neutrophil to lymphocyte ratio in missed abortion. Rev Bras Ginecol Obstet. 2020;42(5):235–9. 10.1055/s-0040-1709693 PMC10316850 32483803

[26] Zhang L, Ma Y, Zhu YN, Shen YF, Li S, Zhu B. Association of RDW-CV, PDW and NLR with recurrent spontaneous abortion. Chin J Clin Lab Sci. 2018;36(6):432–4.

[27] Kaplanoglu M, Yuce T, Bulbul M. Decreased mean platelet volume is associated with the developing stage of fetoplacental unit in spontaneous abortion. Int J Clin Exp Med. 2015;8(7):11301–6. PMC4565322 26379939

[28] Li Q, MA C. Significance of Hcy, folic acid, PLT, MPV and PDW detection in patients with recurrent spontaneous abortion. Int J Lab Med. 2017;38(5):613–4.

[29] Li XJ, Yi H, Huang YL, Liu ZY. The correlation of thromboela-stogram and platelet parameters with recurrent miscarriage. Chin J Clin Obstet Gynecol. 2020;21(6):634–5.

[30] Lu XJ, Liang JM, Seng LL, Bai YQ, Gao JX, Qin LX, et al. Analyze the diagnostic value of different detection methods for recurrent miscarriage. Chin J Lab Diag. 2022;26(4):0480.

[31] Oğlak SC, Aydın MF. Are neutrophil to lymphocyte ratio and platelet to lymphocyte ratio clinically useful for the prediction of early pregnancy loss? Ginekol Pol. 2020;91(9):524–7. 10.5603/GP.a2020.0082 33030732

[32] Qian SJ, Li J, Shen XH. Diagnostic value and clinical significance of combined detection of PDW and NLR in patients with recurrent spotaneous abortion. Int J Lab Med. 2020;41(9):1101–4.

[33] Yilmaz M, Delibas IB, Isaoglu U, Ingec M, Borekci B, Ulug P. Relationship between mean platelet volume and recurrent miscarriage: a preliminary study. Arch Med Sci. 2015;11(5):989–93. 10.5114/aoms.2013.40095 PMC4624730 26528341

[34] Yu PY, Zhao FL, Zhang XH, Zhao LJ, Zhao LY. Predtive values of thromboelastograph, mean platelet volume/platelet count ratio and fibrinogen level in recurrent spontaneous abortion. JJU-ME. 2020;46(1):127–31.

[35] Qin XY, Shen HH, Zhou WJ, Mei J, Lu H, Tan XF, et al. Insight of autophagy in spontaneous miscarriage. Int J Biol Sci. 2022;18(3):1150–70. 10.7150/ijbs.68335 PMC8771834 35173545

[36] Bitsadze V, Khizroeva J, Alexander M, Elalamy I. Venous thrombosis risk factors in pregnant women. J Perinat Med. 2022;50(5):505–18. 10.1515/jpm-2022-0008 35044114

[37] Peshkova AD, Safiullina SI, Evtugina NG, Baras YS, Ataullakhanov FI, Weisel JW, et al. Premorbid hemostasis in women with a history of pregnancy loss. Thromb Haemost. 2019;119(12):1994–2004. 10.1055/s-0039-1696972 31587245

[38] Middeldorp S, Naue C, Köhler C. Thrombophilia, thrombosis and thromboprophylaxis in pregnancy: for what and in whom? Hamostaseologie. 2022;42(1):54–64. 10.1055/a-1717-7663 35196731

[39] Chen Y, Zhong H, Zhao Y, Luo X, Gao W. Role of platelet biomarkers in inflammatory response. Biomark Res. 2020;8:28. 10.1186/s40364-020-00207-2 PMC7397646 32774856

[40] Pala AA, Urcun YS. Is the mean platelet volume a predictive factor for atrial fibrillation developing after coronary artery bypass grafting in elderly patients? Heart Surg Forum. 2020;23(6):E809–14. 10.1532/hsf.3201 33234211

[41] Braester A, Stemer G, Khouri S, Raviv B, Barhoum M. Is there a predictive value of high mean platelet volume in early diagnosis of venous thromboembolism? Isr Med Assoc J. 2021;23(10):635–8. 34672445

[42] Edvardsen MS, Hansen ES, Hindberg K, Morelli VM, Ueland T, Aukrust P, et al. Combined effects of plasma von Willebrand factor and platelet measures on the risk of incident venous thromboembolism. Blood. 2021;138(22):2269–77. 10.1182/blood.2021011494 34161566

[43] Dechanuwong P, Phuan-Udom R. Hematological parameters as a predictor of disease remission in patients with rheumatoid arthritis. Ann Med Surg (Lond). 2021;72:103085. 10.1016/j.amsu.2021.103085 PMC8626573 34868575

[44] Masoumi M, Shadmanfar S, Davatchi F, Shahram F, Akhlagi M, Faezi T, et al. Correlation of clinical signs and symptoms of Behçet’s disease with mean platelet volume (MPV) and red cell distribution width (RDW). Orphanet J Rare Dis. 2020;15(1):297. 10.1186/s13023-020-01588-1 PMC7579941 33087144

[45] Taha SI, Samaan SF, Ibrahim RA, Moustafa NM, El-Sehsah EM, Youssef MK. Can complete blood count picture tell us more about the activity of rheumatological diseases? Clin Med Insights Arthritis Musculoskelet Disord. 2022;15:11795441221089182. 10.1177/11795441221089182 PMC9036329 35481333

[46] Uzkeser H, Keskin H, Haliloglu S, Cayir Y, Karaaslan Y, Kosar A, et al. Is mean platelet volume related to disease activity in systemic lupus erythematosus? Int J Clin Pract. 2021;75(11):e14676. 10.1111/ijcp.14676 34322962

[47] Vagdatli E, Gounari E, Lazaridou E, Katsibourlia E, Tsikopoulou F, Labrianou I. Platelet distribution width: a simple, practical and specific marker of activation of coagulation. Hippokratia. 2010;14(1):28–32. PMC2843567 20411056

[48] Meng J, Wang S, Zhang M, Fan S, Zhang L, Liang C. TP73 G4C14-A4T14 polymorphism and cancer susceptibility: evidence from 36 case-control studies. Biosci Rep. 2018;38(6):BSR20181452. 10.1042/BSR20181452 PMC6294616 30420492

