# Supplementary material for: Relationship between platelet activation markers and spontaneous abortion: A meta-analysis
Source: Open Life Sci. 2022 Dec 15;17(1):1669–78. doi: 10.1515/biol-2022-0485 (PMC9755698; doi:10.1515/biol-2022-0485)
Supplement: Supplementary Material [file biol-2022-0485-sm.pdf]

Supplementary material

PubMed

(((mean platelet volume [Title/Abstract]) OR (MPV[Title/Abstract])) OR ((platelet distribution width [Title/Abstract]) OR (PDW[Title/Abstract]))) AND (((abortion [Title/Abstract]) OR (miscarriage [Title/Abstract])) OR (pregnancy loss [Title/Abstract]))

Embase

#1'mean platelet volume':ab
#2 'MPV':ab
#3 #1 OR #2
#4 'platelet distribution width':ab
#5 'PDW':ab
#6 #4 OR #5
#7 #3 OR #6
#8 'abortion':ab
#9 'miscarriage':ab
#10 ''pregnancy loss':ab
#11 #7 OR #8 OR #9
#12 #7 AND #11

CNKI

(Abstract: 'miscarriage' + 'abortion' + 'pregnancy loss') AND (Abstract: 'mean platelet volume' + MPV + 'platelet distribution width' + PDW)
